# XRT for visualizing microstructure of extruded meat replacers

**DOI:** 10.1016/j.crfs.2023.100457

**Published:** 2023-02-06

**Authors:** Maaike Nieuwland, Walter Heijnis, Atze-Jan van der Goot, Remco Hamoen

**Affiliations:** a Wageningen Food & Biobased Research, Wageningen University & Research, PO Box 17, 6700 AA, Wageningen, the Netherlands; b Food Process Engineering, Wageningen University & Research, PO Box 17, 6700 AA, Wageningen, the Netherlands

**Keywords:** XRT, X-Ray Tomography, SPI, Soy Protein Isolate, HME, High Moisture Extrusion, SPC, Soy Protein Concentrate, IF, Insoluble Fibre, SF, Soluble Fibre, DM, Dry Matter

## Abstract

X-Ray Tomography (XRT) was used to visualize the microstructure of extruded meat replacers. The high moisture extrudates contained lamella, that became visible upon pulling the extrudate apart. In frozen state, these lamella could be visualized with XRT. The freezing increased the density difference between the water-rich and protein-rich layers, thus increasing the contrast obtained in the XRT. Differences in the physical structure were reflected in the measured structure. In non-frozen samples, no lamella were visible, indicating insufficient contrast.

Because of the contrast obtained in frozen samples, we conclude that the XRT technique is a valuable addition to investigate extrudate structure, that can be used to quantify differences in extrudates obtained by for example variation in composition. Here we showed a higher lamella thickness for soy protein isolate (SPI) compared to more fibre-rich soy protein samples.

## Highlights

• XRT is a promising technique for non-invasive visualisation of high moisture extrudates.
• In frozen state, lamella of high moisture extruded meat analogues could be visualized with XRT.
• Structures measured in frozen state with XRT were comparable with lamella observed visually.

## Graphical abstract

**Figure:**
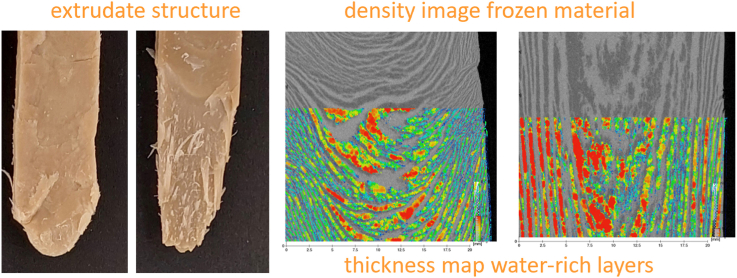

## 1 Introduction

Meat replacers receive a lot of interest as those products are thought to help consumers to achieve a more balanced diet between proteins from animal and plant sources (Aiking and de Boer, 2020). It is thought that the protein transition can be advanced if meat replacers can mimic the good eating quality of meat: the taste, the fibrous and tender texture, and its juiciness (Hoek et al., 2011; Michel et al., 2021). Having such high quality meat replacers will allow consumers to shift to a more plant-based diet without changing their eating habits. The first meat replacers were almost exclusively based on ingredients produced with low moisture extrusion, the ingredients best known as texturized vegetable protein (TVP). In the past years, there is a growing research interest in meat replacers produced via high moisture extrusion (HME) (Cornet et al., 2022; Pietsch et al., 2019). The structure formation in HME, essential for the quality of the meat replacers, is however not fully understood yet. The validity of the various hypotheses concerning the physical/chemical processing occurring during high moisture extrusion often still need to be confirmed, and which is the subject of extensive research currently performed in various groups world-wide (Schreuders et al., 2021, van der Sman et al., submitted). Currently, we hold the hypothesis that microstructure formation in extruded meat replacers happens at three different length scales: at the nanometer scale there is protein alignment (Tian et al., 2020), at the micron scale there is elongation of the dispersed (biopolymer) phases (Dekkers et al., 2018b), and at the millimeter scale there is phase separation or syneresis (formation of a water-rich phase)(Sandoval Murillo et al., 2019; Wittek et al., 2021). The latter structuring process is visible to the eye, which is the characteristic parabolic structure – when breaking extrudates in two.

A key factor to better understand structure formation is the availability of good analytical techniques that can visualize resulting structure formed during the extrusion process. It enables the link between processing parameters and the resulting quality. However, visualisation of these structures is a challenge (Schreuders et al., 2021). An option is to freeze the structures, slice those using a microtome and image these thin slices with microscopy (Wittek et al., 2021). This works if the structure has clear visual differences on a sufficiently large length scale. A disadvantage is the laborous sample preparation. Fluorescence microscopy (CLSM) has been used to obtain two dimensional and in some cases three dimensional images of thin slices of meat replacers (Dekkers et al., 2018a). CLSM has a limited penetration depth and needs a fluorescent dye to penetrate the sample. Ideally a non-invasive technique is used, that reveals the internal structure of the sample. Magnetic resonance imaging (MRI) provides 3D imaged depending on the spin of specific nuclei. The main limitation of this technique is that the resolution is limited to about 100 μm (Wang et al., 2018). Acoustic imaging has a similar resolution and is costly for 3D images (Wang et al., 2018).

X-ray tomography (XRT) is a non-invasive visualisation technique for which no staining or microtome slicing is required. XRT works based on density differences in the sample. It has been used in many applications, ranging from histology to soil research (Porre et al., 2016). The technique is also used in food research to observe salt crystals (Fulladosa et al., 2010), the holes in cheese (Guggisberg et al., 2013) or ice cream (Mo et al., 2018). In fruits, defects can be analyzed (Herremans et al., 2013). In meat replacers, the size distribution and shape of air bubbles is analyzed (Dekkers et al., 2018a). With XRT, density differences of up to 10% can be detected, allowing for a relatively easy distinction of air, water and fat phases in many food products (Dittmann et al., 2016; Metilli et al., 2021; Wang et al., 2018). The contrast between dry material and air is also large, allowing for indirect investigation of ice crystals via freeze-drying (Kobayashi and Suzuki, 2019; Sato et al., 2016; Zhao and Takhar, 2017). However, the density difference between ice crystals (970 kg/m^3^ (van der Sman, 2008),) and organic material (density up to 1350 kg/m^3^) is also sufficient to get an image of ice (Kobayashi and Suzuki, 2019; Vicent et al., 2019). The maximum resolution that can be achieved using XRT is about 1 μm, which serves well to investigate for example dried droplets (Bouman et al., 2016).

In this article, we explore the potential of XRT to investigate internal structure of high moisture extrudates. Freezing enhances the density differences between phases to more than 10%, allowing for visualisation with XRT. During freezing water is extracted from the biopolymer phase or biopolymer phases, so that both the water-rich and biopolymer rich phases approach their bulk densities of respectively 970 and up to 1350 kg/m^3^ (van der Sman, 2008).

## 2 Materials and methods

### 2.1 Materials

In this study commercially available ingredients were used. The soy protein isolates (Supro EX37 HG and Supro500E, SPI) and soy protein concentrate (Alpha 8, SPC) were obtained from Solae LLC (St. Louis, MO, USA). Insoluble soy fibre (Unifiber Soy, IF) was obtained from Vitablend and the soluble soy fibre (SoyaFibe S-CA100, SF) was supplied by Fuji Oil Co, Ltd., Japan.

Soluble and insoluble fibre content of the soy protein concentrate, the Unifiber and the SoyaFibe S-CA100 were measured by Eurofins, using a modification of the protocol AOAC2011.25. The results of the fibre content is shown in Table 1. Based on the fibre content of the ingredients, the fibre content of the seven samples prepared by high moisture extrusion was also calculated and are shown in the table as well.

**Table 1:** Fibre content on dry matter (DM). Total fibre is based on (high molecular weight) insoluble fibre, (high molecular weight) soluble fibre, and low molecular weight fibre. The first three rows are measured fibre content, the next seven rows are calculated based on the measured fibre content on the ingredients.

Sample	Composition	Determination of fibre content by	Insoluble fibre (% DM)	Soluble fibre (% DM)	Total fibre (% DM)
Pure SPC	100% SPC	Measurement	19.46	3.04	24.24
Pure IF	100% IF	Measurement	67.89	5.22	75.78
Pure SF	100% SF	Measurement	<2	65.00	65.00
SPI extrudate	100% SPI	Calculation	0.00	0.00	0.00
SPC + SPI extrudate	52% SPI; 48% SPC	Calculation	9.34	1.46	11.63
SPC extrudate	100% SPC	Calculation	19.46	3.04	24.24
SPI + IF + SF extrudate	66% SPI; 29% IF; 5% SF	Calculation	19.80	4.76	25.23
SPI + SF extrudate	66% SPI; 34% UF	Calculation	<1	22.10	22.10
SPI + IF extrudate	66% SPI; 34% IF	Calculation	23.08	1.78	25.76
SPC + UF extrudate	76% SPC; 24%UF	Calculation	31.08	3.57	36.61

### 2.2 Extrusion experiments

**Fig. 1:**
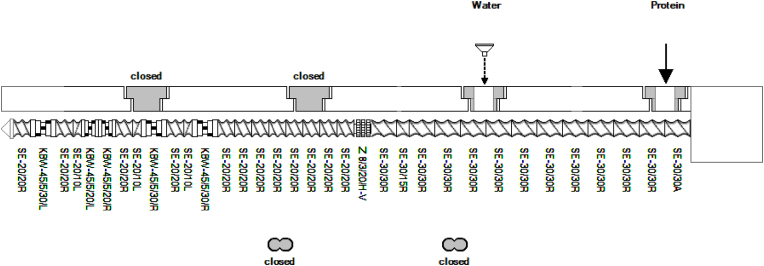
Mounted screw used during extrusion experiments.

The extrusion experiments were performed on a co-rotating twin-screw extruder, TwinLab-F 20/40 D (Brabender GmbH, Duisburg, Germany), with a L/D ratio of 40 and a screw diameter of 20 mm. A 300 mm segmented cooling die was attached, consisting of three 100 mm cooling die pieces with a 25 × 7 mm geometry. The mounted screw is depicted in Fig. 1 and consists of four kneading sections. The protein/powder inlet is at 0D and the water inlet at 10D. The extruder barrel can be heated in four different zones.

Solids were dosed via a gravimetric feeder (DDSR20 – free-fall; Brabender GmbH, Duisburg, Germany) at the inlet located at 0D. The water was dosed via a peristaltic pump (Watson Marlow 120U; Watson-Marlow Limited, Cornwall, UK) at the inlet located at 10D.

In these experiments the extrusion settings were kept constant. The screw speed was set at 800 rpm and the feed was set at 4.5 kg/h. The ratio of solid and liquid feed was adjusted on the determined moisture content of the blend to achieve an overall moisture content of 55% (w/w). The heating zones were set at 40 °C; 80 °C; 110 °C and 130 °C. The temperature of the cooling die was set at 50 °C. The extrudates were cut on a roller band and further analyzed by XRT.

### 2.3 XRT analysis

A GE Phoenix v|tome|x m tomographer (General Electric, Wunstorf, Germany) was used for 3D non-invasive and non-destructive imaging Unless mentioned otherwise, a frozen sample was placed in a styrofoam shell and cooled using an air stream of −20 °C air to prevent the sample from defrosting. The system contains two X-ray sources. The 240 kV micro focus tube with tungsten target was employed. X-rays were produced with a voltage of 120 kV and a current of 120 μA. The measurements were done once for each sample composition.

The images were recorded by a GE Dynamic 41|200 detector array with 2024 × 2024 pixels (pixel size 200 μm). The detector is located 815 mm from the X-ray source. The object was placed 78.1 mm from the X-ray source. This result in a spatial resolution of 20 μm. A full scan consist of 1500 projections over 360°. The 1st image was skipped. The saved projection is the average of 3 images where every image is obtained over 83 ms exposure time.

GE reconstruction software (Wunstorf, Germany) was used to calculate the 3D structure via back projection.

Next to the seven extrudates, an extrudate with an added layer of pure ice was measured, to compare the grey value of the water-rich layer to that of pure water. The added ice showed the same grey value as the water-rich layers in the extrudate.

### 2.4 Image analysis

The 3D images, obtained using the v|tome|x XRT and the GE reconstruction software, were analyzed using Avizo imaging software Avizo3D 2021.2. A thickness map routine was performed to get a measure for lamella thickness. The thickness map is defined as the largest diameter that fit around the voxel in the selected volume. It represents the local size of the structure.

## 3 Results

Seven samples, based on soy protein, were created with high moisture extrusion. Soy protein isolate (SPI) was mixed with soluble and insoluble soy fibre (respectively SF and IF), and compared with SPC and with SPC with added insoluble fibre. The fibre content ranged between 0% fibres based on dry matter (DM) (SPI), and 37% DM (soy protein concentrate (SPC) with extra added insoluble fibre (IF)), see Table 1.

**Fig. 2:**
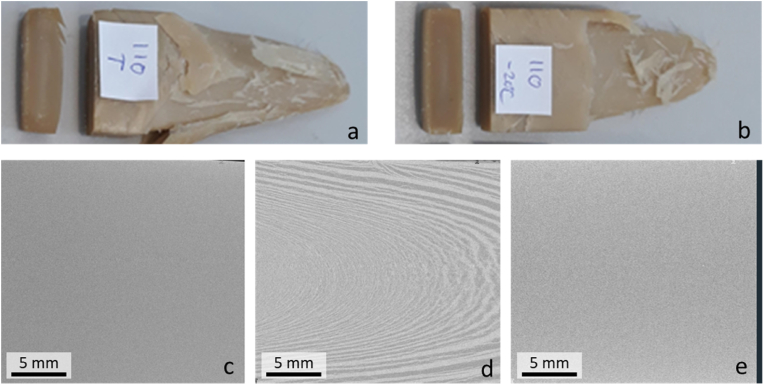
Effect of freezing on wedge. A) picture of fresh sample; B) picture of defrosted sample; C) X-Ray reconstruction of a fresh sample; D) X-Ray reconstruction of a frozen sample; E) X-Ray reconstruction of a defrosted sample.

Extruded samples were cut in a length wise direction, which revealed a homogeneous structure. Still, when pulled apart in the direction of the extrusion, a parabolic wedge formed (Fig. 2). After freezing and defrosting, the wedge upon pulling the sample apart is similar as in a fresh sample (Fig. 2a and b). This observation is true for all extruded samples with fibre present. The sample prepared from pure SPI was very tough and therefore hard to pull apart.

Despite the wedge being visible upon pulling the extrudate apart, in an XRT scan, a fresh sample showed a homogeneous density distribution of a structure without bubbles or density differences at the image resolution (20 μm) (Fig. 2c). After freezing, layers became visible in the structure when imaged with XRT (Fig. 2d). Defrosting of this sample resulted in an image that was homogeneously grey again (Fig. 2e). Therefore, we have used frozen samples to study the lamellar structures of the seven high moisture extrudates. The formation of ice crystals in the freezing process can change the structure of the product, even irreversibly. However, here freezing mostly made the product structure more clear, while not changing it in an irreversible manner. For example, the material showed a similar wedge upon pulling apart before and after freezing. Furthermore, the structure visible in XRT measurements is similar to the wedge structure observed at non-frozen state by pulling apart the structure.

The structure seen in the frozen sample was explained by the existence of multiple phases: ice and one or multiple biopolymer phases. The ice phase is consisting of pure water, as shown by the additional measurement of pure water that showed the same grey value as the water-rich phase. In this study, no distinction was made between the different polymer phases, and for ease of reference they are depicted as ‘the protein-rich phase’.

**Fig. 3:**
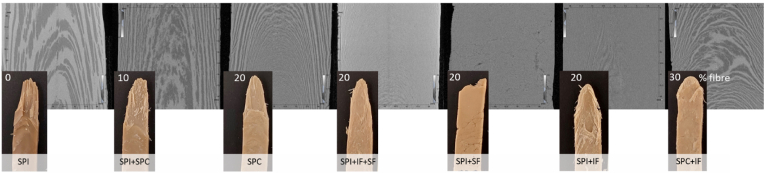
Wedges and X-Ray reconstructions of seven samples of soy protein with varying fibre content.

After defrosting, the samples were pulled apart to show the wedge structure (Fig. 3). These pictures show that there is a difference in wedge structure depending on the ingredient composition used. SPI gives a tough and rubbery structure that is hard to pull apart and has a less layered wedge. Mixing with soluble fibre yields a brittle and sticky structure without a wedge. For the samples containing SPC, toughness increased with an increase in fibre content. The wedge length observed in the X-Ray reconstructed images qualitatively corresponded well to the wedge length seen in the extrudates. Long wedges in the SPI + SPC were visible, while a homogeneous structure was observed in case of SPI + IF. This demonstrates that the images made with XRT can be used to quantify the sample structure.

**Fig. 4:**
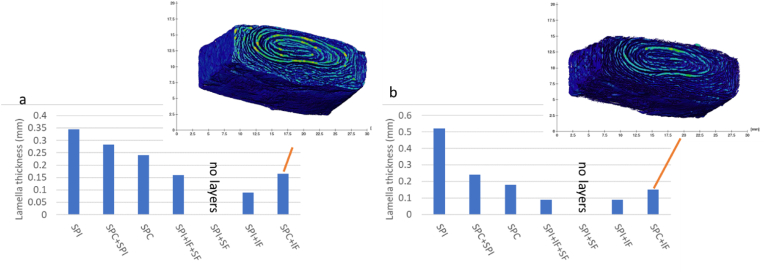
Thickness of a) the protein-rich and b) the water-rich layers of the extrudates. An example of the thickness maps for sample SPC + IF is shown. Sample SPI + SF cannot be reliably investigated as the structure is basically homogeneous.

To quantify the structure, first the protein-rich, denser, lighter grey value part of the sample was selected, to estimate the size of the lamella. Subsequently, the water-rich, less dense and thus darker part of the image was selected, and the lamella size was studied as well. This yields a thickness distribution of the protein-rich and water-rich lamella. In Fig. 4 the median layer thickness (thickness at 50% of the histogram) is shown for the protein-rich and water-rich layers. It is clear that SPI has the thickest layers for both protein-rich and water-rich domains. With increasing fibre content the thickness of both protein and water layers decreased. The type of fibre and the type of starting material is thus a determining factor in the final structure. Addition of only soluble fibre to SPI does not yield a lamella structure at all, while insoluble fibre added to SPI yields very thin lamella.

## 4 Discussion

Fresh samples show a homogeneous microstructure in the XRT image. Upon freezing, a layered microstructure becomes visible that disappears again upon defrosting. The frozen wedge structures observed with XRT qualitatively match the wedge structures obtained after pulling the defrosted extrudates apart. This reversible visibility of layers can be explained by the ice crystal formation during the freezing process. When the sample is cooled down, ice is formed first in the water-rich layers, and in the freezing process the ice crystallisation extracts water from the protein-rich phase. The ice phase consists of pure water, and solutes (including proteins) are excluded. Consequently, the protein-rich layers get more concentrated. The larger density difference created by water freezing that allows for visualisation in XRT stems both from the lower density of ice compared to water and from the difference in proteins and solutes in the protein-rich phase. The factors that are important in the formation of layers and that govern their thickness and the distribution over the phases are not fully elucidated. A number of hypothesis on the roles of different ingredients and the processes on formation of a fibrous phase are described in the recent review by Van der Sman et al. (submitted). Further investigation is necessary to support these hypothesis.

## 5 Conclusions

The use of XRT is a promising tool to better understand the structure formed during extrusion. Here, we demonstrated that samples in frozen state can reveal differences in microstructure and XRT measurements allow quantification of the wedge length and lamella thickness. Samples have to be frozen to create contrast between low density ice crystals and a high density protein phase. In non-frozen samples, we did not find sufficient contrast. The wedge length found via XRT corresponded well with wedges observed when pulling apart non-frozen samples.

The wedge structure formed upon extrusion depends on processing conditions and ingredient choice. In this article a number of ingredients were varied, and it was shown that with XRT differences in the structure could be quantified. Further research is necessary to understand the structural changes better.

## CRediT authorship contribution statement

**Maaike Nieuwland:** Software, Validation, Formal analysis, Investigation, Writing – original draft, Writing – review & editing, Visualization. **Walter Heijnis:** Conceptualization, Investigation, Writing – review & editing. **Atze-Jan van der Goot:** Writing – review & editing. **Remco Hamoen:** Methodology, Software, Validation, Writing – review & editing, Visualization.

## Declaration of competing interest

The authors declare that they have no known competing financial interests or personal relationships that could have appeared to influence the work reported in this paper.

## Data Availability

Data will be made available on request.
